# NetAP-ML: Machine Learning-Assisted Adaptive Polling Technique for Virtualized IoT Devices

**DOI:** 10.3390/s23031484

**Published:** 2023-01-29

**Authors:** Hyunchan Park, Younghun Go, Kyungwoon Lee, Cheol-Ho Hong

**Affiliations:** 1 Division of Computer Science and Engineering, Jeonbuk National University, Jeonju 54896, Republic of Korea; 2 School of Electronics Engineering, Kyungpook National University, Daugu 41566, Republic of Korea; 3 School of Electrical and Electronics Engineering, Chung-Ang University, Seoul 06974, Republic of Korea

**Keywords:** edge computing, I/O virtualization, adaptive polling, machine learning

## Abstract

To maximize the performance of IoT devices in edge computing, an adaptive polling technique that efficiently and accurately searches for the workload-optimized polling interval is required. In this paper, we propose NetAP-ML, which utilizes a machine learning technique to shrink the search space for finding an optimal polling interval. NetAP-ML is able to minimize the performance degradation in the search process and find a more accurate polling interval with the random forest regression algorithm. We implement and evaluate NetAP-ML in a Linux system. Our experimental setup consists of a various number of virtual machines (2–4) and threads (1–5). We demonstrate that NetAP-ML provides up to 23% higher bandwidth than the state-of-the-art technique.

## 1. Introduction

Edge computing plays a crucial role in wireless systems for the Internet of Things (IoT), as it reduces the message latency of IoT devices and maximizes the throughput by locating the computing resources near IoT devices rather than cloud data centers [1,2]. This is because various IoT services such as mobile video streaming, virtual reality, and augmented reality require high speed network processing with an utrahigh success rate and minimal latency [3,4]. For maximizing the throughput and minimizing the latency of such devices, polling, a traditional mechanism to continuously monitor the status of the devices, has been utilized instead of an interrupt-based mechanism for a non-virtualized environment [5,6,7]. As many edge computing systems adopt server virtualization to manage hardware resources effectively [8,9], it is also required to apply the polling mechanism to existing virtualization software.

However, there are the following issues regarding applying polling in edge computing that adopts virtualization: First, as existing virtualization software implements an interrupt-based notification method rather than polling, a significant modification of the network processing path in the virtualization software is necessary. Second, when we conduct polling periodically, the optimal polling interval may change dynamically depending on the characteristics of various workloads.

To address the first issue, we proposed NetAP [10] previously, which applies adaptive polling to the network processing path in a virtualized environment. In the original KVM, packets generated by a virtual machine (VM) are sent to the virtual network device in the KVM hypervisor or the host system, which is implemented with shared memory between the VM and the host. When the number of packets generated from the VM exceeds a certain threshold, the virtual network device is notified through a virtual IRQ (vIRQ). However, this vIRQ mechanism results in expensive VM-Exit and VM-Entry operations and decreases the network performance [11]. NetAP first replaced this interrupt-based mechanism with adaptive polling in which the hypervisor periodically inspects packet arrivals every polling interval.

To address the second issue, NetAP proposed a technique to find a near-optimal polling interval that maximizes network performance by using golden-section searching [12]. Different workloads on multiple VMs exhibit various characteristics regarding the frequency of packet generation and packet size. The hypervisor then needs to allow a different polling interval for each workload to maximize the performance. The golden-section searching algorithm finds a near-optimal polling interval for each workload by obtaining the maximum or minimum value within a given range of a unimodal function [12]. To the best of our knowledge, NetAP is the only framework to provide a solution for finding an optimal polling interval in KVM-based virtualization.

However, NetAP has a significant limitation. As the golden-section searching algorithm is based on trial and error, it can degrade the performance because of incorrect attempts in the intermediate search process. The algorithm first assumes that there is an optimal interval that provides maximum performance within a certain interval range. Then, it tests several interval values within the range and tries to find the optimal interval by narrowing the range, while searching for the new polling interval, the performance can be severely affected by an inappropriate polling interval. Thus, the user applications on the VMs would suffer from degraded and unstable performance during the search process. In summary, golden-section searching takes a lot of time to find an optimal interval because of the broad search space, and during search time, the the network performance can be degraded due to an incorrect attempt. Therefore, we need to develop an efficient technique to reduce the search space for the golden-section searching algorithm.

In this paper, we propose NetAP-ML, which employs machine learning (ML) to provide a more efficient and accurate searching technique to find a near-optimal polling interval for maximizing network performance. The key idea is to shrink the search space by using a trained model with ML. First, we generate a dataset that contains the relationship between each interval and performance for various workloads. Then, we train random forest regression, one of the popular machine learning-based regression techniques, to predict a polling interval approximate to the near-optimal interval. Second, NetAP-ML shrinks the search space using the predicted value and searches for a near-optimal value through golden-section searching. As the search space is reduced, it is possible to find a near-optimal polling interval that exhibits high performance more accurately and without performance degradation. We implement and evaluate NetAP-ML on a KVM-virtualized Linux system and demonstrate that NetAP-ML provides up to 23% higher bandwidth than NetAP.

The main contribution of this article compared with the previous work, NetAP, is summarized as follows: NetAP applies golden-section searching, but it searches every polling interval from 50 to 4000 ms. This search process takes much time and can degrade the network performance as it can produce an inefficient polling interval during the search process. NetAP-ML adopts a random forest regression model to predict a near optimal value for given VMs. Then, NetAP-ML shrinks the search space of golden-section searching around the predicted value. Therefore, NetAP-ML can reduce the search time and improve the network performance as the produced interval during the search process is close to the optimal value.

The remainder of this article is structured as follows: In Section 2, we explain the background of VirtIO and NetAP. Section 3 elaborates on the design of NetAP-ML. Section 4 shows the performance evaluation results. Section 5 explains related work. Finally, we present our conclusions in Section 6.

## 2. Background

This section describes the architectures and behaviors of VirtIO and NetAP as background knowledge.

### 2.1. VirtIO

Our previous work, NetAP [10], is based on virtualized systems based on KVM that utilizes VirtIO modules [13,14] for I/O (i.e., network and block device) virtualization. VirtIO modules deliver the I/O requests between user applications running in a guest OS and the I/O devices on the host via the VirtIO front-end device driver. The VirtIO front-end device driver for the network runs on the guest OS, which is represented as a virtual network device. When a user application in the guest OS transmits network packets, the front-end driver receives the packets through the network stack of the guest OS. Then, the front-end driver stores the packets in shared memory to deliver them to the host. The shared memory is built for data communication between the corresponding VM and the host. To notify the host OS of the packet delivery, the front-end driver generates a vIRQ once, even for multiple packets, to minimize the overhead of vIRQ processing.

In the host OS, a virtual host (vhost) thread conveys the packets from the shared memory to the network stack of the host OS when the vIRQ is acknowledged. KVM assigns a vhost thread for each VM, and the vhost thread deals with the I/O requests of the corresponding VM. The notification method of the vhost thread incorporates the traditional polling mechanism and an interrupt-based notification. The vhost thread first fetches consecutive packets stored in the shared memory using a polling mechanism when it acknowledges the vIRQ for the first time. Then, it processes and transmits the packets through corresponding network devices. Similarly, packet reception is performed in the opposite direction.

The major overhead of I/O processing in virtualized environments resides in the vIRQ processing. This is because the host OS receives the vIRQ by waking up the sleeping vhost thread. As a result, the vIRQ processing brings frequent VM-Exit and VM-Entry operations that alter the execution mode in the CPU, which leads to a significant virtualization overhead [6,11,15].

### 2.2. NetAP

NetAP uses a periodic polling technique in the vhost thread to maximize the network and CPU utilization. In NetAP, the modified vhost thread polls the shared queue periodically instead of waiting for a vIRQ from the guest OS. At each polling interval, the vhost thread processes packets sent from the guest OS. Between the intervals, the guest OS generates packets and puts them into the shared queue. At the next interval, the vhost thread processes the buffered packets in the shared queue. This packet processing is repeated during the execution of the workload. This polling mechanism prevents the VM-Exit and VM-Entry operations that consume a lot of CPU cycles. Therefore, NetAP can improve network performance with efficient CPU use.

The most important part of NetAP operation is the polling interval; while giving the guest OS time to produce enough packets, the network device should not be idle for long periods. An optimal polling interval is closely related to the characteristics of the workload executing in the guest OS. NetAP outperforms VirtIO when a polling interval that is optimized for each workload is found.

However, the problem of finding the optimal polling interval is challenging for the following reasons: First, the characteristics of the workload running on each guest OS change dynamically. Second, multiple guest OSes running on the same host system influence each other. NetAP uses the golden-section searching algorithm to solve this problem.

The golden-section search technique searches the maximum value for the target function f(x) is as follows [10]: First, we configure two x-coordinates $x_{l}$ and $x_{u}$ to search. We assume that the maximum value is between $x_{l}$ and $x_{u}$. Second, we choose $x_{1}$, where $x_{l}<x_{1}<x_{u}$ and the $\left(x_{u}-x_{1}\right)/\left(x_{u}-x_{l}\right)$ is *R*, a reciprocal of the golden ratio (R≈0.618). These three x-coordinates are golden-section triplets. Third, we set the new x-coordinate, $x_{2}$, where $x_{l}<x_{2}<x_{u}$ and the $\left(x_{2}-x_{l}\right)/\left(x_{u}-x_{l}\right)$ is *R*. Then, we evaluate the $f(x_{l})$, $f(x_{1})$, $f(x_{2})$, and $f(x_{u})$. If $f\left(x_{2}\right)<f\left(x_{1}\right)$, then $x_{l}$, $x_{1}$, and $x_{2}$ are chosen as new triplets, otherwise if $f\left(x_{2}\right)>f\left(x_{1}\right)$, then $x_{1}$, $x_{2}$, and $x_{u}$ are chosen. Then, this process is iterated until the outer x-coordinates are closer than ϵ which is the predetermined minimal distance to terminate the algorithm. The complexity of the algorithm is logarithmic because it requires only one additional evaluation for each iteration. The big-O notation of the algorithm is shown in Equation (1), where *N* is the number of iterations required to find the solution [16,17].

(1) $$N=O(log\frac{1}{\epsilon })$$

After applying the pre-configured minimum and maximum polling intervals for each guest OS, the optimal polling interval between the two values is found using golden-section searching. For this purpose, NetAP tries 11 polling interval candidates in golden-section searching. As it takes about one second per each candidate to evaluate network performance, NetAP requires more than 10 s to obtain the optimal interval per workload.

Nevertheless, NetAP achieved a network performance improvement of up to 31.16% while using only 32.92% of CPU time compared to VirtIO. Therefore, if an appropriate polling interval is found quickly, high efficiency can be achieved for virtualized I/O processing. The remaining problem is to find the polling interval faster and more accurately.

## 3. Design

This section first describes the overall architecture of NetAP-ML. Then, we explain how we develop ML-Predictor, which is a key component of NetAP-ML.

### 3.1. NetAP-ML Architecture

**Figure 1:**
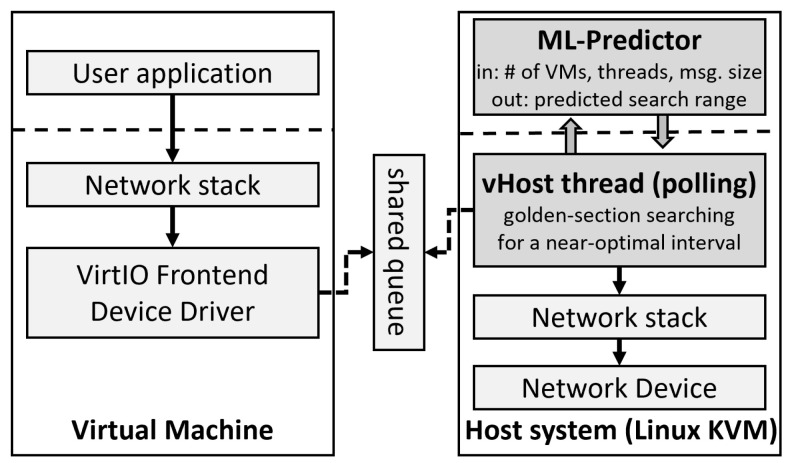
Architecture of NetAP-ML.

NetAP-ML operates as following: (1) it uses an ML technique to obtain a narrow range of search space and (2) it performs golden-section searching on the corresponding range to find a near-optimal polling interval. Figure 1 shows the components of NetAP-ML related to the I/O request processing path in the KVM-based virtualized system.

When a user application in the VM requests a packet transfer from KVM, the request is stored in the shared queue through the VirtIO front-end device driver; VirtIO is the main framework for I/O virtualization in KVM. In original KVM, after the packet is buffered in the shared queue by the front-end driver, VirtIO generates a vIRQ for the host system, so that the vhost thread in charge of the corresponding virtual NIC (vNIC) can perform packet handling. The vhost thread handles and delivers the packet to the physical NIC through the network stack of the host system. Then, the vhost thread polls for a while to efficiently process upcoming packets.

NetAP excludes the vIRQ mechanism and only employs periodic polling. This means that the vIRQ is not raised and the packets from the user application in the VM are buffered in the shared queue. The packets are periodically handled by the vhost thread in KVM every polling interval. Thus, the overhead caused by vIRQ is eliminated, and the processing performance is improved. At the same time, in order to minimize CPU waste incurred by continuous polling, NetAP adopts adaptive polling and optimizes the polling interval using golden-section searching for various workloads that exhibit a dynamic change in incoming packet rate.

In NetAP-ML, a user-level component called the ML-Predictor is developed in the host system to obtain a narrow range of search space. The ML-Predictor receives information required for prediction, such as the number of VMs/threads and the message size of the currently running workloads, from the vhost thread. The vhost thread operates in kernel mode and the user-level ML-Predictor communicates through the file system. Then, the trained model in the ML-Predictor predicts a near-optimal polling interval for each workload based on the input data. ML-Predictor configures a narrow search space around the interval (e.g., the predicted interval ± 500 ms). The range is provided to the vhost thread. At last, golden-section searching is invoked based on the informed range. NetAP-ML allows golden-section searching to operate more efficiently through this search range adjustment. In our experiments, NetAP-ML successfully finds the optimized polling interval even when the search range is only reduced to a quarter of NetAP’s search space.

In summary, NetAP-ML and NetAP use the same golden-section searching, but the range for searching is different. NetAP wants to support a variety of workloads using a fixed, wide search range. This makes the golden-section searching slower and less accurate. In contrast, NetAP-ML uses ML-Predictor to set a narrow search range for each workload. This increases the speed and accuracy of golden-section searching. In conclusion, NetAP-ML minimizes performance degradation by making the process of finding the optimal polling interval more efficient.

### 3.2. Machine Learning Model

To build an ML-trained model in the ML-Predictor, we considered four representative ML algorithms: linear regression (LR), adaptive boost regression (ABR), kernel ridge regression (KRR), and random forest regression (RFR). We particularly selected algorithms for regression analysis because in our prior work, we found that there is a linear relation between the polling interval and the network throughput of workloads in the VMs.

LR is one of the most widely used approaches for regression analysis, which models a linear relation between two independent variables. Furthermore, we utilized ABR, which is one of the ensemble methods and is based on the boosting algorithm. Next, KRR utilizes ridge regression that estimates the coefficients of multiple-regression models to learn a linear function. At last, RFR constructs a group of numerical decision trees where each decision tree is grown using the given training set and a random vector independently. This offers high performance and accuracy as it resolves the over-fitting problem of the decision tree algorithm by reducing the over-fitting of datasets.

For training, we collected real-world data from our evaluation system. A total of 6400 bandwidth results were collected by combining the following factors, which are highly related to the network performance: VMs = {1,2,3,6}, threads = {1,2,3,6}, packet sizes in bytes = {64,128,256,512,1024}, and polling intervals = 50–4000 ms with a step of 50 ms.

Using the collected data, we trained the four respective models with hyperparameter tuning using a randomized grid search. We utilized scikit-learn [18], an open-source Python library, equipped with state-of-the-art ML tools. For each set of hyperparameters, we performed five-fold cross-validation on the training set. This means that we separated the training set into five smaller sets and generated a prediction error on each set after fitting the model to the other four sets, then calculated the validation estimate as to the average of the prediction errors. Furthermore, we divided the entire data into the training set and the validation set. Note that the training set of data is only for training LR, ABR, KRR, and RFR, while the validation set was utilized for measuring the accuracy.

**Figure 2:**
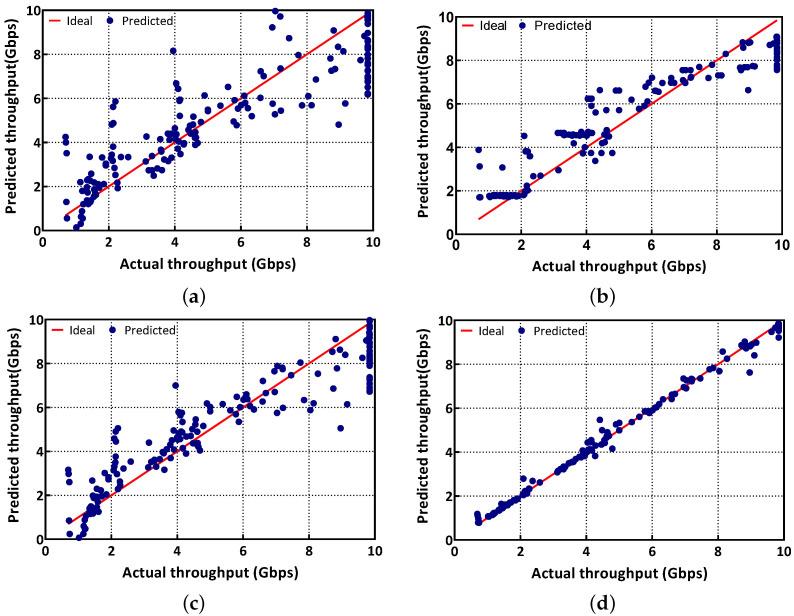
Test data results that show the actual value on x-axis and the predicted value (on the y-axis) using (**a**) LR, (**b**) ABR, (**c**) KRR, and (**d**) RFR.

Figure 2 illustrates the predicted network throughput (y-axis) using LR, ABR, KRR, and RFR, respectively, in the validation set of data. Note that the x-axis shows the actual throughput in the validation set, and the red line indicates the ideal case where the predicted throughput is identical to the actual throughput. Thus, the blue dots close to the red line mean that the model offers high accuracy. First, Figure 2a shows that many blue dots lie away from the red line, which indicates the low accuracy of LR. The results from ABR and KRR in Figure 2b,c depict that the blue dots are closer to the red line compared to those of LR. However, ABR and KRR still show some wrong predictions. For example, both ABR and KRR have difficulty in predicting the throughput close to the link rate (i.e., 10 Gbps) and infer lower throughput than the actual throughput. On the other hand, when we utilize RFR, the model shows the best predictions that is identical to the actual throughput, as shown in Figure 2d. This indicates that RFR offers the highest accuracy among the four models.

In addition, we calculated the root-mean-square error (RMSE) that indicates how concentrated the data is around the line of best fit in predictions for various data points into a single measure of predictive powerby Equation (2) for each model. $y_{predict}$ indicates the predicted value using each model, while $y_{real}$ indicates actual values.

(2) $$\mathrm{RMSE}=\sqrt{{\left(y_{predict}-y_{real}\right)}^{2}}$$

The RFR offers the lowest RMSE of 0.21, while the RMSE of LR, ABR, and KRR are 1.49, 1.08, and 1.21, respectively. The reason that RFR offers high accuracy is that RFR overcomes the limitations of the decision tree algorithm by controlling the over-fitting of the dataset. RFR trains a group of numerical decision trees on sub-samples of the dataset. As each group of decision trees utilizes different datasets for training, it has randomness, which can resolve over-fitting issues. By evaluating each group of decision trees, RFR examines important features in the dataset, which have a large impact on the prediction. This improves the overall model accuracy of RFR compared to other models that we evaluated. Moreover, RFR requires a short training time compared to other complex machine learning techniques such as reinforcement learning. For example, it only takes less than two minutes to train 900 samples on a desktop PC equipped with Intel i7-10700K CPU@3.8 GHz.

Although the results of RFR were promising, ML techniques cannot completely replace golden-section searching yet. Several other factors cannot be fully addressed by RFR, such as changes in the CPU clock or randomly generated interrupts. Thus, instead of using the predicted interval obtained by the ML-trained model directly, NetAP-ML sets a narrow search space range based on the predicted interval.

## 4. Evaluation

We implemented NetAP-ML on Linux v5.1.5 and evaluated it to demonstrate that NetAP-ML searches a near-optimal polling interval more efficiently and accurately than the previous work, NetAP. Experiments were performed on a KVM-virtualized system equipped with a 24-core CPU, 128 GB RAM, and a 10 GbE NIC. A VM is allocated six vCPUs and 1 GB of memory. The NIC is shared with VMs using MacVtap. We ran the Netperf benchmark in each VM, which communicates with a separate Netperf server system using TCP, and measured the aggregated network bandwidth in Gbps on the host system.

We performed a prediction of the near-optimal polling interval using RFR with the following three cases:

- C1: 3 VMs, 2 threads, and 128 B message size;
- C2: 2 VMs, 5 threads, and 96 B message size;
- C3: 4 VMs, 1 thread, and 192 B message size.

Those cases were selected as they can clearly reveal the performance change according to the predicted polling interval. The training dataset includes the same factors of C1 (i.e., 3 VMs, 2 threads, and 128 B message size), but it does not contain the factors of C2 and C3. Thus, we can determine how good the prediction of RFR was from the results for C2 and C3.

**Figure 3:**
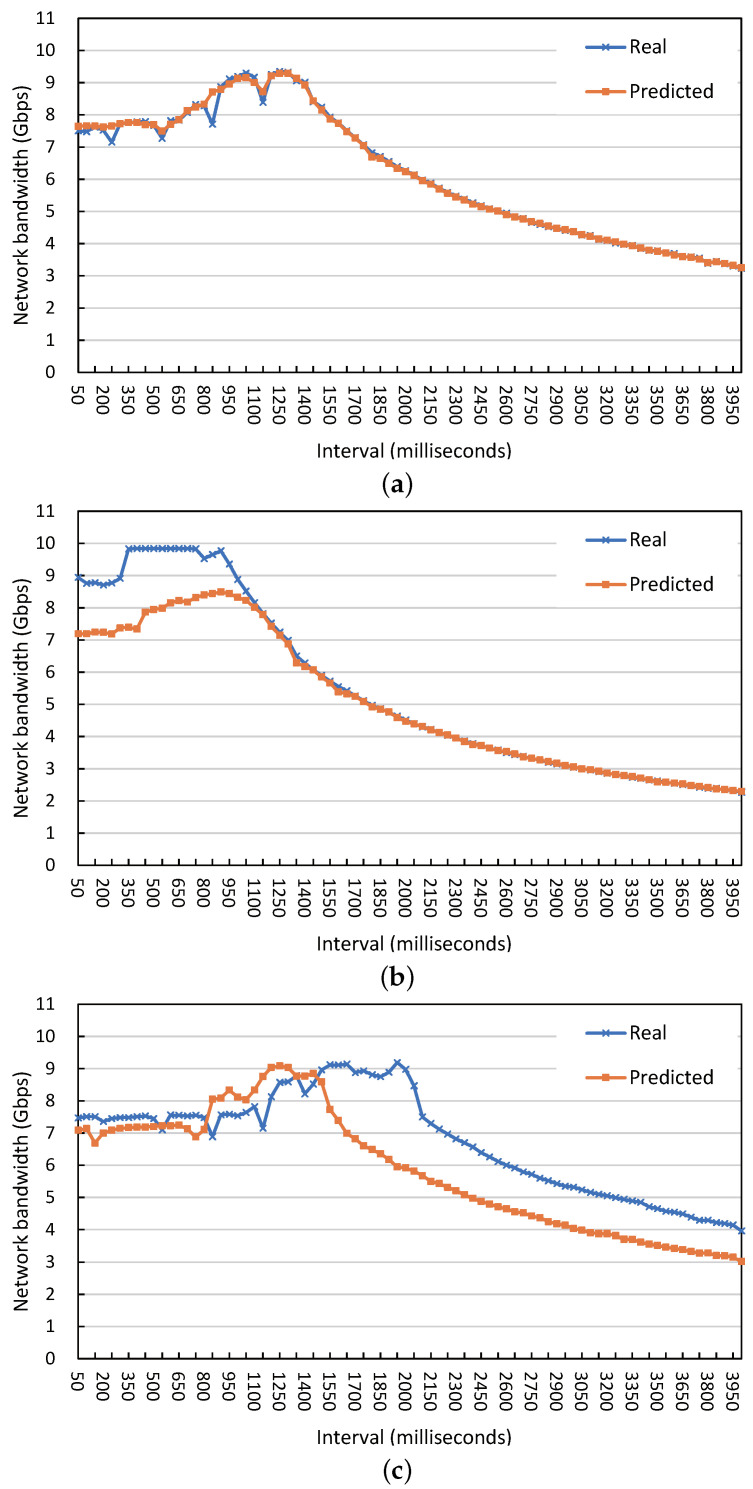
Experimental and predicted performance according to the polling interval. (**a**) 3 VMs, 2 threads, and 128 B message size. (**b**) 2 VMs, 5 threads, and 96 B message size. (**c**) 4 VMs, 1 thread, and 192 B message size.

Figure 3 shows the performance prediction results using our ML-Predictor for the three cases and the actual results in the real hardware, according to the polling interval. For C1, the predicted and actual results are very similar, and C2 also generally shows a similar pattern. In particular, the predicted optimal interval does not deviate from the actual optimal interval. The predicted and measured optimal intervals were 1250 ms and 1251 ms for C1, respectively. C2 showed a maximum performance of from 400 ms to 800 ms as a result of actual measurement, and the predicted optimal interval was 876 ms. In the case of C3, the results from prediction and actual measurements were slightly different while showing a slight overlap ranging from 50 ms to 1400 ms. The predicted and measured optimal intervals for C3 were 1950 ms and 1225 ms, respectively.

Then, we evaluated the performance of NetAP-ML for searching the optimal polling intervals to maximize network performance with regard to efficiency and accuracy compared to NetAP. The main difference between NetAP and NetAP-ML is the size of the search space. In the case of NetAP, the search space ranges from 100 ms to 4100 ms, while that of NetAP-ML is ±500 ms of the predicted interval from the ML-Predictor. Thus, the search range of NetAP is four times larger than that of NetAP-ML.

We made Netperf run for 30 s for each case. We employed a fixed default interval (2100 ms, a median of the NetAP’s search range) during the first 10 s, and afterwards we performed adaptation with golden-section searching from 10 to 20 s. During the last 10 s, we utilized the last found near-optimal interval without change. Each case was performed 10 times, and the performance was observed at 2 s intervals.

**Figure 4:**
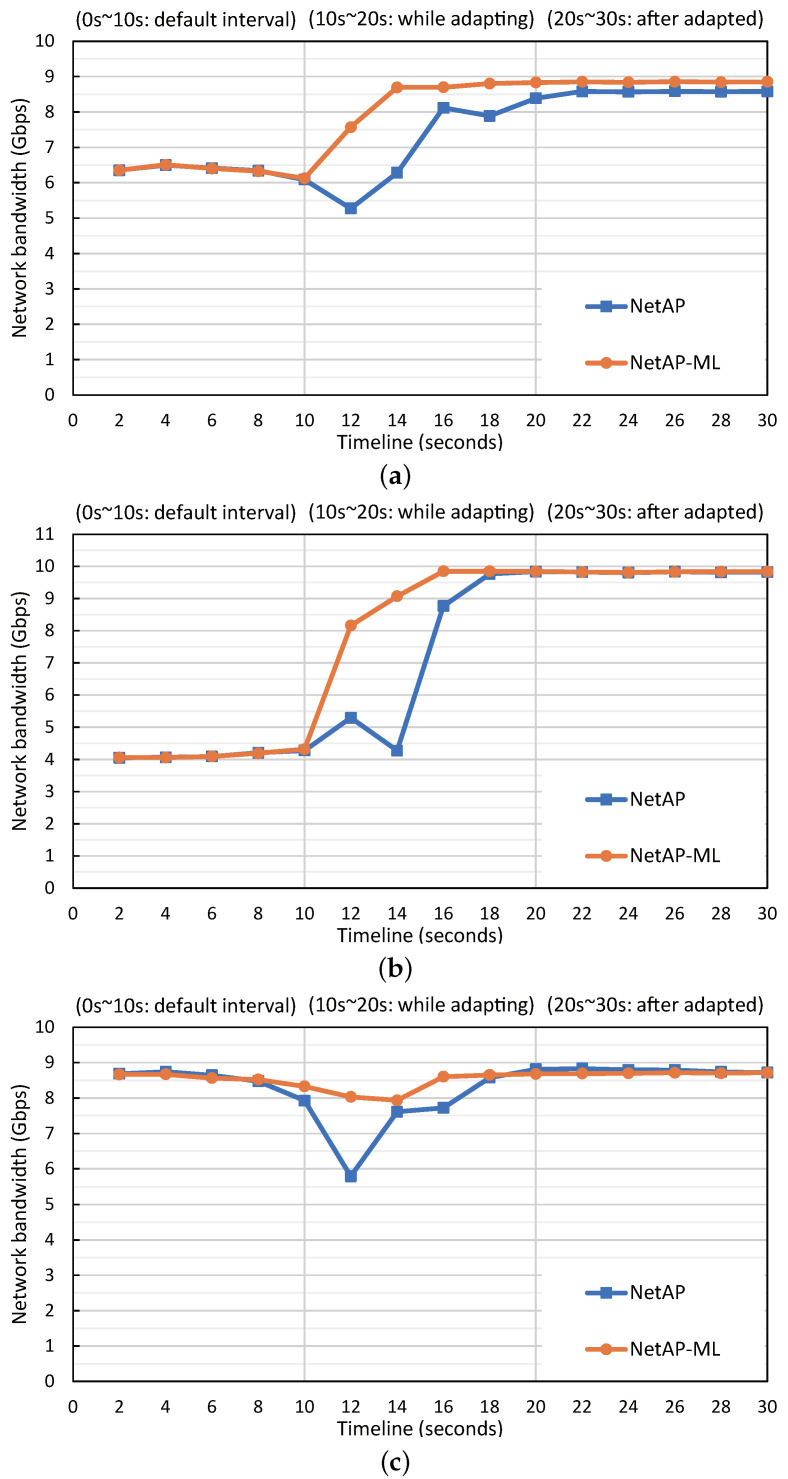
Performance changes during and after adaptation of NetAP and NetAP-ML. (**a**) 3 VMs, 2 threads, and 128 B message size. (**b**) 2 VMs, 5 threads, and 96 B message size. (**c**) 4 VMs, 1 thread, and 192 B message size.

Figure 4 depicts the performance changes for each case, and Table 1 shows the mean and standard deviation values for each section (i.e., during and after). Note that ’during’ indicates the time in the middle of adaptation which is from 10 s to 20 s. Furthermore, ’after’ means from 20 s 30 s, where the optimal polling interval is applied. In the case of C1, NetAP-ML shows better performance than NetAP both during and after adaptation. In particular, C1 improves the performance by 18% on average during adaptation and 3% after adaptation. The standard deviation also shows a 48% reduction during adaptation compared to NetAP.

In the case of C2, the performance improvement of NetAP-ML during adaptation is similar, but the performance after adaptation is 23% higher than NetAP. At this time, the standard deviation decreased by 71%. In the case of C3, although the prediction was not performed well, it succeeds in finding a polling interval that shows a similar level of performance to that of NetAP within the set range. Furthermore, the performance improved by 9% and the standard deviation reduced by 52% during adaptation.

These results indicate that NetAP-ML searches for the optimal polling interval stably with little performance degradation even in a narrow search space, owing to the predicted value being close to the optimal interval. Although accurate prediction achieves better performance, a slightly wrong prediction can be overcome in the current set range of ±500 ms. If a prediction result becomes more accurate, the search space would be further reduced to show better network performance and shorten the adaptation time.

## 5. Existing Approaches

We divide previous studies relevant to NetAP-ML into two categories: (1) adaptive polling that aims to improve traditional polling or interrupt-based I/O processing and (2) ML-based approaches that enhance the resource allocation in host systems.

### 5.1. Adaptive Polling for Efficient I/O Processing in Virtualized Environments

The limitations of the interrupt-based and polling-based techniques have been studied for a long time, including non-virtualized and virtualized environments. For the non-virtualized environments, several studies [19,20,21,22,23,24] proposed to change the period of polling operations or the mode of interrupt processing depending on the amount of traffic. Even though the studies showed an improvement in performance, it is difficult to apply them to virtualized environments. This is because the non-virtualized environment does not consider the I/O virtualization, such as VirtIO modules. Moreover, there are other factors that should be considered for network performance in virtualized environments, such as the period of performance monitoring and the change in the packet-processing speed of the VM. Therefore, it is necessary to conduct more in-depth research to optimize the polling intervals to maximize the network performance in virtualized environments.

For virtualized environments, previous studies [25,26,27,28] mostly adopted two approaches: utilizing hardware support or bypassing OS intervention in network processing. As recent network interface cards (NICS) support virtualization technologies inside the device, there have been several attempts to adopt the new devices. Even though such hardware-assisted approaches bring high performance, they have limitations, such as high costs of installing new devices and lack of flexibility. This is because the hardware-assisted techniques assign PCI addresses to VMs, which makes the live migration of VMs difficult [29]. The other approach, that bypasses OS intervention in network processing, also achieves high network performance by delivering network packets directly to VMs from NICs. The advantage of the bypass approach is that the technique can be employed without hardware support by installing new software or device drivers [27]. However, when the network packets bypass kernel-level inspection, they potentially face security problems. This is because the memory where the packets are stored is exposed to user space while security features are almost absent [30].

### 5.2. ML-Based Approaches for Resource Allocation

Recently, many studies [31,32,33,34,35] have been conducted to adopt ML/DL techniques to enhance resource allocation or performance monitoring for diverse applications in cloud data centers.

First, Firm [31] and Autopilot [34] suggested utilizing reinforcement learning to improve resource allocation in cloud data centers. Reinforcement learning is a representative unsupervised learning technique that explores action space and generates optimal policies. Previous studies design reinforcement learning models that determine a proper amount of computing resources for allocation to minimize performance degradation. Even though these techniques improve end-to-end latency of cloud applications and resource utilization, they require a considerable amount of training to provide high accuracy. For example, Firm needed to train the model for more than an hour to outperform existing techniques.

Second, the efficiency of performance management can be improved by predicting the performance of applications in advance. Several studies [32,33,35] utilized LSTM [36], which receives sequences of data and is well-suited to classifying, processing, and making predictions based on time series data. For example, Seer [33] found the pattern of application performance in time was based on the performance history. Evaluation results in Seer show that the LSTM-based model provides high accuracy in predicting the performance degradation of applications, which can improve the responsiveness of performance management.

Even though previous studies adopting ML/DL techniques require a significant training time and a sufficient amount of data for training, they show promising results in reducing performance degradation. NetAP-ML adopts random forest regression to reduce the search space of previous NetAP through in-depth research, which is different from other studies [21] that only propose to adjust polling interval linearly.

## 6. Conclusions

In this paper, we proposed NetAP-ML, which uses ML to find the optimal polling interval to maximize the performance of virtualized high-speed I/O devices. NetAP-ML utilizes ML to reduce the search space so that (1) it minimizes the performance degradation in the search process and (2) it finds a polling inverval closer to the optimal value than our previous work NetAP. To achieve these goals, we first generated a data set that presents the relationship between each polling interval and performance for various workloads. Then, we trained an ML model to predict a near-optimal polling interval. At last, NetAP-ML configures the narrowed search range around the interval and uses golden-section searching in the narrowed range to find the optimal polling interval for the workload currently running.

We implemented NetAP-ML in a virtualized Linux system and demonstrated the effectiveness of NetAP-ML through experiments for various cases. The results show that NetAP-ML improves the bandwidth by up to 23% more than NetAP, the state-of-the-art method which uses golden-section searching with a wide search range for finding the optimal polling interval.

The important contribution of this paper is the application of the ML technique to increase the performance of virtualized I/O devices. In future research, (1) it is necessary to devise a new ML model trained with the experimental results derived from NetAP and NetAP-ML and (2) it is necessary to apply the model to more diverse and large-scale systems and further develop it through feedback. Ultimately, we should aim for a technique that can track and reflect the characteristics of workloads in real-time and dynamically modify polling time for I/O processing accordingly.

## Figures and Tables

**Table 1:** Average and standard deviation (in Gb/s) for the second experiment.

		Average		Standard Deviation	
**Test Case**		**NetAP**	**NetAP-ML**	**NetAP**	**NetAP-ML**
**C1**	**During**	7.19	8.52 (+18%)	1.24	0.64 (−48%)
	**After**	8.58	8.85 (+3%)	0.47	0.43
**C2**	**During**	7.59	9.36 (+23%)	2.34	0.67 (−71%)
	**After**	9.82	9.83	0.05	0.04
**C3**	**During**	7.70	8.38 (+9%)	1.12	0.54 (−52%)
	**After**	8.78	8.70	0.38	0.34

## Data Availability

Data available on request.
